# Minieditorial: Quercetina Melhora o Perfil Lipídico e Apolipoproteico em Ratos Tratados com Glicocorticoides em Altas Doses

**DOI:** 10.36660/abc.20200461

**Published:** 2020-07-28

**Authors:** Bruna Paola Murino Rafacho

**Affiliations:** 1 Faculdade de Ciências Farmacêuticas, Alimentos e Nutrição Universidade Federal de Mato Grosso do Sul Campo GrandeMS Brasil Faculdade de Ciências Farmacêuticas, Alimentos e Nutrição (FACFAN) da Universidade Federal de Mato Grosso do Sul, Campo Grande, MS – Brasil

**Keywords:** Quercetina, Antioxidantes, Anti-Inflamatórios, Flavonoides, Ratos, Glicocorticoides

*Minieditorial referente ao artigo: Quercetina Melhora o Perfil Lipídico e Apolipoproteico em Ratos Tratados com Glicocorticoides em Altas Doses*

Flavonoides, tais como antocianinas, flavonóis, flavanóis, flavanonas, flavonas e isoflavonas, são os polifenóis mais abundantes na dieta humana. A quercetina, um tipo de flavonol, é um dos mais estudados entre esses compostos.^1 - 3^ A quercetina é um metabólito secundário de planta, da subclasse flavonol da família dos flavonoides presentes em muitas frutas e vegetais, como por exemplo maçãs, uvas, cebolas e pimentas.^3 , 4^

Nos últimos anos, as evidências mostraram que a quercetina é um potente produto natural antioxidante e anti-inflamatório.^4^ A quercetina é capaz de proteger as células contra danos oxidativos causados por espécies reativas e ativar enzimas antioxidantes, como a heme oxigenase e o fator nuclear eritroide 2 relacionado ao fator 2 em diferentes modelos.^5 - 7^ Em relação à saúde cardiovascular, estudos *in vitro* , em animais e em seres humanos, relataram efeitos benéficos, incluindo a melhora do perfil lipídico,^4 , 5 , 8 - 10^ conforme explorado no estudo de Derakhshanian et al. apresentado nesta seção.

O estudo de Derakhshanian et al.,^11^ aborda o efeito da quercetina na hipercolesterolemia induzida por altas doses de metilprednisolona em ratos, um novo uso desse composto bioativo.^11^ Glicocorticoides (GC), como a metilprednisolona, são amplamente utilizados no tratamento de diferentes doenças. No entanto, altas doses de GC podem levar a efeitos adversos, incluindo alterações no metabolismo lipídico.^12^ Os autores testaram duas doses de quercetina por seis semanas e obtiveram uma redução no colesterol total (CT), lipoproteína de baixa densidade colesterol (LDL), triglicérides (TG) e lipoproteína de alta densidade colesterol (HDL), razões CT/HDL, TG/HDL e LDL/HDL e apolipoproteína B (apo B)/apolipoproteína (A1), um indicador do equilíbrio aterogênico plasmático^13^ e um potencial marcador de risco cardiovascular.^14^ Os autores também discutem que pouco se sabe sobre os mecanismos pelos quais os GCs alteram os níveis de lipídios no sangue, sugerindo que o efeito da quercetina pode ser atribuído à sua propriedade antioxidante e ao potencial de modulação da glicose.^11^ Um ponto interessante do trabalho de Derakhshanian et al.,^11^ é que ambas as doses produziram um efeito protetor na hipocolesterolemia induzida por glicocorticoides, excluindo um efeito superior da dose mais alta empregada. É importante observar que pesquisas anteriores sobre estimativa de consumo humano variam de 3 a 40mg no padrão de dieta ocidental a 250mg na dieta rica em frutas e vegetais.^3^ Portanto, a dose mais baixa poderia ser obtida de uma dieta rica em fontes de quercetina, como apontado pelo presente artigo.^11^

O interesse em compostos naturais para o manejo de diferentes condições aumentou nos últimos anos devido ao seu potencial de segurança em comparação aos compostos sintéticos.^4^ Embora ainda existam muitas dúvidas sobre o uso de flavonoides na saúde humana, o estudo de Derakhshanian et al.,^11^ adiciona dados sobre o papel adjuvante da quercetina nos distúrbios metabólicos.
