# Supplementary material for: Minieditorial: Quercetina Melhora o Perfil Lipídico e Apolipoproteico em Ratos Tratados com Glicocorticoides em Altas Doses
Source: Arq Bras Cardiol. 2020 Jul 28;115(1):109–10. [Article in Portuguese] doi: 10.36660/abc.20200461 (PMC8384330; doi:10.36660/abc.20200461)
Supplement: Translation

# Translation: Short Editorial: Quercetin Ameliorates Lipid and Apolipoprotein Profile in High-Dose Glucocorticoid Treated Rats

**Authors:** Bruna Paola Murino Rafacho

**Affiliations:** 1 Brazil Faculdade de Ciências Farmacêuticas, Alimentos e Nutrição (FACFAN) da Universidade Federal de Mato Grosso do Sul, Campo Grande, MS – Brazil

**Keywords:** Quercetin, Antioxidants, Anti-Inflamatórios Agents, Flavonoids, Rats, Glucocorticoids

*Short Editorial related to the article: Quercetin Ameliorates Lipid and Apolipoprotein Profile in High-Dose Glucocorticoid Treated Rats*

Flavonoids, such as anthocyanins, flavonols, flavanols, flavanones, flavones, and isoflavones are the most abundant polyphenols in the human diet. The flavonol quercetin is one of the most studied among these compounds.^1 - 3^ Quercetin is a secondary plant metabolite of the flavonol subclass of flavonoids present in many fruits and vegetables, e.g. apples, grapes, onions and peppers.^3 , 4^

In the past years, evidence showed that quercetin is a potent antioxidant and anti-inflammatory natural product.^4^ Quercetin is able to protect cells from oxidative damage caused by reactive species and activate antioxidant enzymes, such as heme oxygenase and nuclear factor erythroid 2–related factor 2 in different models.^5 - 7^ Regarding cardiovascular health, in vitro, animal and human studies have reported beneficial effects, including improving lipid profile,^4 , 5 , 8 - 10^ as explored in the study of Derakhshanian et al. presented in this section.

The study by Derakhshanian et al.^11^ addresses the effect of quercetin on hypercholesterolemia induced by high doses of methylprednisolone in rats, novel use of this bioactive compound.^11^ Glucocorticoids (GC) such as methylprednisolone are widely used in the treatment of different diseases. However, high dose GC can lead to adverse effects, including changes in lipid metabolism.^12^ The authors tested two doses of quercetin for six weeks and obtained a reduction in total cholesterol (CT), low-density lipoprotein cholesterol (LDL), triglycerides (TG) and high-density lipoprotein cholesterol (HDL), CT/HDL, TG/HDL and LDL/HDL ratios and Apolipoprotein B (Apo B)/ Apolipoprotein (A1) ratio, an indicator of plasma atherogenic balance^13^ and a potential cardiovascular risk marker.^14^ The authors also discuss that little is known about the mechanisms by which GCs alter blood lipids, and suggest that the effect of quercetin could be attributed to its antioxidant property and glucose-modulating potential.^11^ An interesting point of Derakhshanian et al.^11^ work is that both doses produced a protective effect on glycosteroid-induced hypocholesterolemia, excluding a superior effect of the higher dose employed. It is important to note that previous research about human intake estimative ranges from 3 - 40mg in Western diet pattern to 250mg in high fruit and vegetable diet.^3^ Thus, the lowest dose could be obtained from a diet rich in quercetin sources, as pointed by the present paper.^11^

The interest in natural compounds for the management of different conditions has grown in recent years due to their safety potential in comparison to synthetic compounds.^4^ Although there are still many questions about the use of flavonoids in human health, the study by Derakhshanian et al.^11^ adds data about the adjuvant role of quercetin in metabolic disorders.
